# Use of Implantable Loop Recorders to Unravel the Cause of Unexplained Syncope

**DOI:** 10.1016/s0972-6292(16)30606-4

**Published:** 2013-03-07

**Authors:** Aniket Puri, Rohit Kumar Srivastava

**Affiliations:** 1 Associate Professor, Dept of Cardiology, Chhatrapati Shahuji Maharaj Medical University, Lucknow, India; 2 PhD scholar, Dept of Physiology, Chhatrapati Shahuji Maharaj Medical University, Lucknow, India

**Keywords:** Syncope, implantable loop recorder

## Abstract

Syncope is a symptom of many underlying disease states, which range from the relatively benign to the life threatening. There are numerous investigations done for patients with recurrent unexplained syncope which may have very low yield when it comes to making a definitive diagnosis. Recently, the implantable loop recorder (ILR) for continuous monitoring of the cardiac rhythm has been launched in India. This review will briefly discuss these current available strategies and focus on the usefulness of an ILR in the definitive diagnosis and treatment of patients with a recurrent unexplained syncope.

## Introduction

Syncope is an abrupt and transient loss of consciousness (TLOC) associated with loss of postural tone that follows a sudden fall in cerebral perfusion. Syncope is a symptom of many underlying disease states, which range from the relatively benign to the life threatening [1]. At assessment, patients are often asymptomatic and the diagnosis is unclear, culminating in frequent hospitalization and resulting in expensive and often repeated investigations, most of which are inconclusive [2].

Syncope accounts for approximately 3% of emergency room visits and 1-6 % of hospital admissions. The prevalence of syncope increases with age from 0.7% in men aged 35-44 to 5.6% in men over the age of 75. In long- term care institutions, the annual incidence is approximately 6 % [3]. The elderly represent the population at greater risk for most causes of syncope.

Once it has been established that the patient has true syncope, it is useful to further classify into 4 categories: 1) reflex neurally mediated; 2) cardiac; 3) orthostatic hypotension; or 4) unexplained. The major causes of syncope are enumerated in Table 1.

Investigation of patients with recurrent unexplained syncope may include electrocardiography, ambulatory Holter monitoring, treadmill exercise testing, neurologic testing, tilt table testing and electrophysiological testing. Recently, a new device, an implantable loop recorder (ILR) has been developed for continuous monitoring of the cardiac rhythm to unravel the cause of unexplained syncope. This review will briefly discuss these current available strategies and focus on the usefulness of an ILR in the definitive diagnosis and treatment of patients with a recurrent unexplained syncope.

More commonly, a focused initial evaluation of syncope leads to a suspected diagnosis, which needs to be confirmed by directed testing. If a diagnosis is confirmed by specific testing, treatment may be initiated. On the other hand, if the diagnosis is not confirmed, then patients are considered to have unexplained syncope. The strategy of evaluation varies according to the severity and frequency of the episodes. The majority of patients with single or rare episodes probably have neurally mediated syncope and tests for confirmation are usually not necessary. If it is not clear that it was syncope, the term 'transient loss of consciousness' (TLOC) is preferable and reappraisal is warranted.

## Approach to assess unexplained syncope

History, physical examination, and electrocardiography are the core of the investigations of syncope with a combined diagnostic yield of 50%. Neurological testing is rarely helpful unless additional neurological signs or symptoms are present and the diagnostic yield for electroencephalography, computed tomography, and Doppler ultrasound is only 6% [4].

### Clinical history and physical examination

History should focus on postural symptom, palpitations, family history and should include the use of medication particularly in the elderly patients. Physical examination should focus on orthostatic blood pressure, cardiac murmurs and specific cardiac disorders e.g. aortic or mitral stenosis. The clinical features suggestive of a particular cause of syncope are enumerated in Table 2.

### Cardiac diagnostic approach

#### Electrocardiography

Electrocardiography is essential in the work-up of patients with unexplained syncope but may reveal a direct cause in only 5 % of patients. Pre-excitation patterns, a long QT- interval, the recently reported Brugada syndrome and characteristic features in patients with arrhythmogenic right ventricular dysplasia should all be considered [5-7]. Table 3

#### Echocardiography

No studies have been specifically designed to assess the usefulness of echocardiography in syncope. However, in patients known to have or suspected of having heart disease, patients suspected of having arrhythmias, echocardiography is an important initial step in diagnostic testing. Unsuspected findings on echocardiography are reported in only 5% to 10% of un-selected patients [8]. This yield is similar to that of 12-lead electrocardiography. The cost-effectiveness of echocardiography in diagnosing the cause of syncope has yet to be determined. In patients with exertional syncope, echocardiography should be done first to exclude hypertrophic cardiomyopathy.

#### Exercise Testing

Exercise stress testing can be used for the evaluation of exertional syncope to diagnose ischemia or exercise-induced tachyarrhythmias or to reproduce post exertional syncope. In one population study of patients with syncope, the yield of the exercise stress test was less than 1% [9]. Data is scarce to determine the yield for ischemia or exercise-induced tachyarrhythmias or to define the test's usefulness in diagnosing exercise-associated syncope. Exercise stress testing is recommended if patients have exercise-associated syncope and if the results of clinical evaluation suggest ischemic heart disease.

#### Tilt table testing

Tilt table testing has emerged as a safe and effective method of identifying individuals with a susceptibility to neurally mediated syncope [10]. Tilt table testing may be performed alone or with pharmacological provocation using isoprenaline or nitrate preparations. The tilt table test is best considered for patients with suspected neurally mediated syncope but in whom the cause is not obvious or in patients with syncope of otherwise unknown origin with no evidence of heart disease. The major problem with tilt table testing is quantifying the test's sensitivity. The sensitivity of the test has been calculated at between 20% and 75% [11]. In those known to have structural heart disease in which electrophysiological studies have not given a diagnostic clue, tilt table testing may prove cost-effective by avoiding expensive and unrewarding neurological investigations such as computed tomography or electroencephalography which might otherwise be requested. Interestingly, the frequency of syncope decreases following a positive test regardless of therapeutic intervention. The test may educate the patient to recognize the warning signs of syncope and to make appropriate changes in posture. [12,13]

#### Holter monitoring

A 24-hour Holter monitor or inpatient telemetry is recommended when symptoms suggest arrhythmic syncope with brief loss of consciousness, no prodrome, palpitations with syncope and in patients who have syncope of unexplained cause, heart disease, or an abnormal electrocardiogram.

### Neurological diagnostic approach

**Figure 1:**
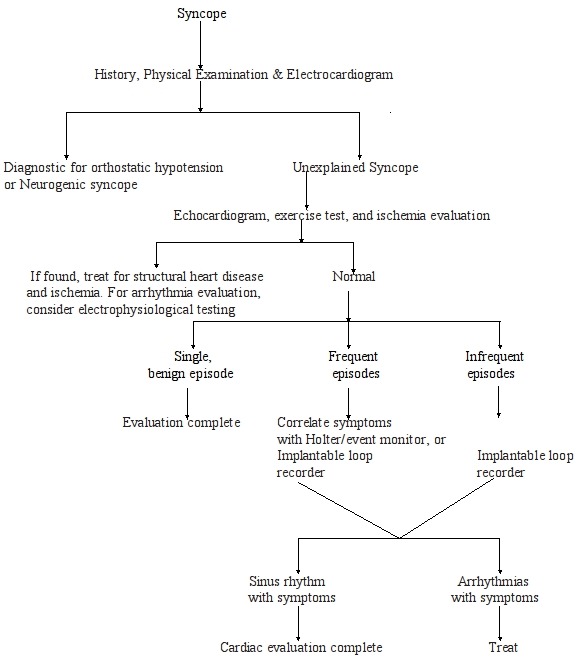
Flow chart for the diagnostic approach to the patient with syncope using an implantable loop recorder

Neurologic tests used for patients with syncope include electroencephalography, brain imaging (computed tomography or magnetic resonance imaging), and neurovascular studies (carotid and transcranial doppler ultrasonographic studies). To determine which patients may benefit from neurologic testing, physicians should take a particularly careful neurologic history for example, patients should be asked about a history of seizure activity, prolonged loss of consciousness, diplopia, headache, and post ictal symptoms and perform a thorough, focused physical examination including a search for bruits or focal neurologic signs. A diagnostic algorithm and approach to a patient with syncope is suggested in Figure 1.

## New method of monitoring to diagnose syncope: "Implantable loop recorder" (ILR)

**Figure 2:**
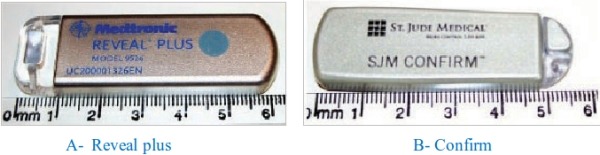
Two type of ILR, the REVEAL PLUS device (MEDTRONIC, Minneapolis, USA) is about 62 x 19 x 8 mm in size and the CONFIRM device (ST JUDE, St Paul, USA) , it is about 56.3 x 18.5 x 8 mm in size

In spite of a detailed screening of patients and often multidisciplinary investigation, more than one third of the patients may remain undiagnosed [14]. Recently, an implantable loop recorder (ILR) has been developed for continuous monitoring of the cardiac rhythm to unravel the cause of unexplained syncope. The ILR is an implantable device that has a solid state loop memory capable of storing electrocardiographic events up to 40 minutes before and 1 to 2 minutes after activation. The ILR has built in electrodes on the back of the device to detect patient's cardiac rhythm and does not require any intra cardiac leads. This device is very small and is typically implanted subcutaneously in the left pectoral region as an out-patient procedure [15]. Currently, there are 2 FDA-approved ILRs available for clinical use as shown in Figure 2. The REVEAL PLUS device (MEDTRONIC, Minneapolis, USA) was the first approved device, it is about 62 x 19 x 8 mm in size and the battery of the device generally lasts 18 months. The CONFIRM device (ST JUDE, St Paul, USA), it is about 56.3 x 18.5 x 8 mm in size and its battery is expected to last up to 3 years. Data are retrieved and analyzed with a compatible programmer but this device also has a remote real-time monitoring capability that allows patients to send data directly to their health care providers.[16]

## Method of Implantation

After identification of the most appropriate pectoral position to record an electrocardiographic lead with a prominent QRS wave, the loop recorder is subcutaneously implanted snugly in a pocket similar to that of a pacemaker implantation, the size of a little finger. After suture of the skin, the quality of electrocardiographic recording is tested in supine and standing positions and during movements of both the arms. The device is explanted after a diagnosis is obtained or if syncope did not recur. The first experiences with the ILR have shown that the device was safe and was able to detect cardiac arrhythmias in about 23-42% of patients. The population in these studies was, however, not systematically evaluated prior to the ILR implantation to reach a diagnosis. [17]

## Clinical evaluation of the ILR

In an initial study of 24 patients, 52% presented with an arrhythmic cause with the vast majority of patients having bradycardia. Treatment was directed at the underlying cause in the 18 patients who received a specific diagnosis. During follow up, syncope did not occur in 16 of the18 treated patients. In 2 patients who underwent explantation of the device after the end of battery life, no recurrence was seen with a final analysis extending the follow-up to 40 ± 10 months[18]. A larger series of 85 patients with recurrent undiagnosed syncope despite extensive evaluation had patients who were eligible if they had at least two syncopal episodes within the previous 2 months or if they had a single syncope with a history of presyncope. In all patients ILR was implanted which resulted in detection of abnormal rhythms in 21 patients, the vast majority having a bradycardia. Interestingly, in 29 patients no arrhythmia was detected despite symptoms. Patients with syncope were more likely to record an arrhythmia during symptoms compared to patients with a history of presyncope (70 % vs 24 % p = 0.005)[19]. Recently another large and prospective study was carried out to collect information on the use of the REVEAL (MEDTRONIC, Minneapolis, USA) ILR in the patient care pathway and to investigate its effectiveness in the diagnosis of unexplained recurrent syncope in everyday clinical practice. Eligible patients had recurrent unexplained syncope or pre-syncope. Follow up was until the first recurrence of a syncopal event leading to a diagnosis or till the end of 1 year. In the course of the study, patients were evaluated by an average of three different specialists for management of their syncope and underwent a median of 13 tests. Average follow-up time after ILR implant was 10±6months. The percentages of patients with recurrence of syncope were 19%, 26% and 36% after 3, 6 and 12 months respectively. Of 218 events within the study, ILR-guided diagnosis was obtained in 170 cases (78%), of which 128 (75%) were of cardiac origin.[20] The findings support the recommendation in current guidelines that an ILR should be implanted early rather than late in the evaluation of unexplained syncope.

Recently we conducted a similar work and enrolled 20 patients of unexplained syncope, out of which 11 patients completed a 12 month, follow up. The diagnostic yield was 100% in the 11 patients among which 7 patients got permanent pacemaker implantation and 4 patients having no arrhythmic event noted with symptoms and 9 patients continue in follow up. [21]

## Therapy Guided by ILR

Little is known about the outcome after ILR-guided specific therapy. In the East Bourne Syncope Assessment Study [22], performed in a typical unselected population, there was an increased diagnostic rate in the group of patients randomized to ILR management and ECG-directed treatments than in conventional investigation group. Despite that a specific ILR directed therapy could be applied to only a minority of patients, the long-term follow-up demonstrated a significant reduction in syncopal events with improved quality of life with ILR based treatment. Since prolonged asystole was the most frequent finding at the time of syncope, pacing was the specific therapy mostly used in the ILR population. In a pooled data of 720 ILR patients from 4 studies, a pacemaker was implanted in 17% of patients, with 45% of those having an ILR-documented event. In the ISSUE 2 study, the 1-year burden of syncope decreased from 0.83±1.57 episodes per patient per year in the control group of patients without any ILR-guided specific therapy to 0.05±0.15 episodes per patient per year in the patients treated with a ILR-guided specific therapy of pacemaker (87% relative risk reduction; P=.001). [23] In another study, after the insertion of a cardiac pacemaker, syncope burden decreased from 2.17 per year to 0.45 per year in 1A or 1B group syncope patients (P=.02) and from 4.57 per year to 0 per year in the type 1C syncope patients (P=.001). Nevertheless, syncope still recurred in 12% (range, 3%-18%) of the patients during the long-term follow up (2.0-3.6 years), especially in those patients more likely to be affected by neurally mediated syncope, accounting for the coexistence of some vasodepressor reflex that cannot be overcome by pacing. Implantable cardioverter-defibrillator and radiofrequency catheter ablation were also consistently used in a few selected patients with ILR documented ventricular and atrial tachyarrhythmias in 1.5% and 3.0% of the patients, respectively [23].

## Limitation of the device

Limitations of the device include the inability to monitor blood pressure changes, the necessity for surgical implant, cost of the device and a small risk of infection. Most patients in whom an arrhythmia is not documented during syncope have either hypotensive syndromes or psychogenic syncope. Hypotensive syndromes include vasovagal syncope, orthostatic hypotension, postprandial hypotension, and vasodepressor carotid carotid sinus hypersensitivity. A facility to track blood pressure behavior in addition to heart rate during symptoms would undoubtedly advance real-time hemodynamic monitoring with implantable devices. The inability of the present device to detect hypotension is a limitation and in the near future the devices capable of recording other physiological parameters may further increase the diagnostic yield [24]. With increasing healthcare costs, a proper selection of patients for implantation of an ILR is mandatory. Total costs of the investigation of patients with recurrent syncope are high because of extensive diagnostic testing [25]. However in a cost analysis study it was demonstrated that ILR implantation could reduce costs as was seen in a pilot study of 24 patients referred for recurrent syncope [26]. There may also be a small risk of interference from electronic article surveillance devices and found that interference may occur causing malfunction of this device [27].

## Conclusion

The prognosis of syncope ranges from benign to life threatening situations and risk stratification should be based on the result of history, physical examination, electrocardiography, and selected noninvasive test. ILR is a novel and useful diagnostic tool in patients with unexplained syncope. ILR is easy to implant and explant and is based on electrocardiography which is useful to establish if there is an arrhythmic cause of syncope, especially when symptoms are recurrent but too infrequent for conventional monitoring techniques.

## Figures and Tables

**Table 1:**
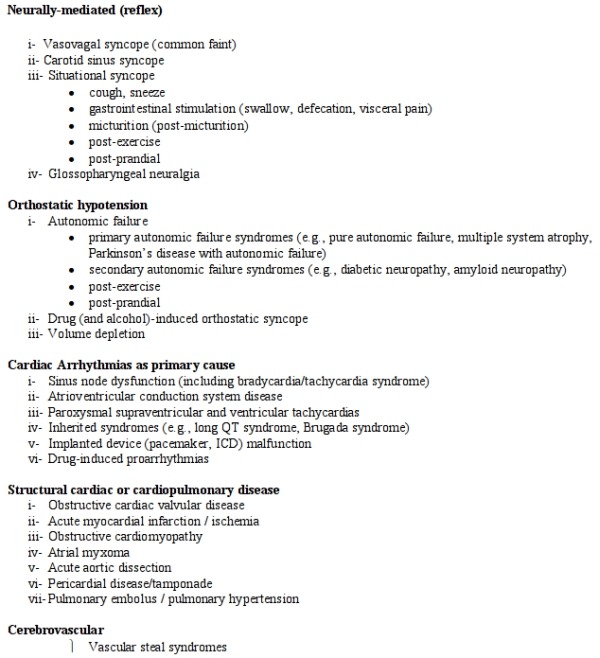
Different causes of syncope

**Table 2:**
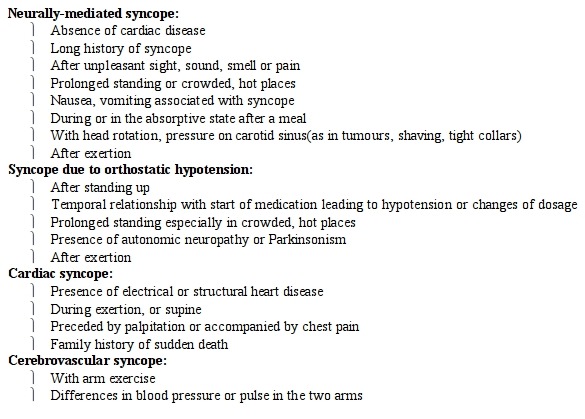
Clinical features suggestive of specific causes of syncope

**Table 3:**
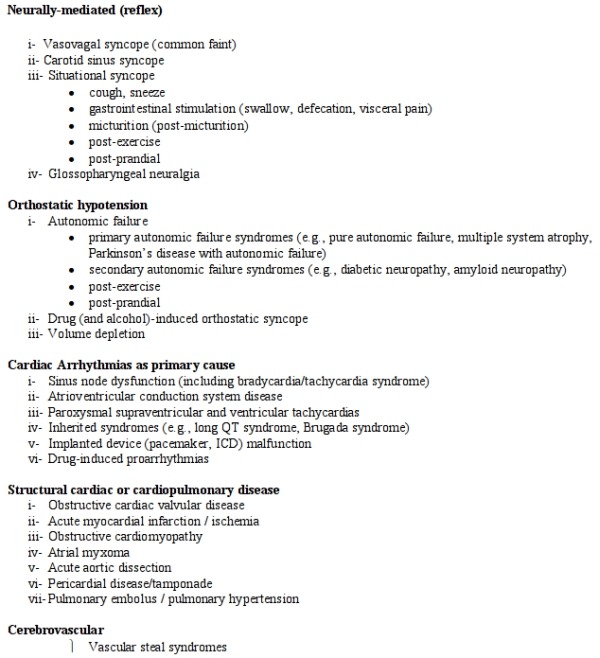
ECG abnormalities suggesting an arrhythmic syncope

**Table 4:**
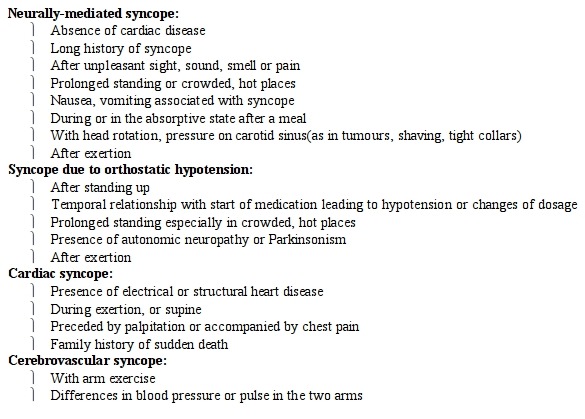
Summary of Recommendations
